# Response of membrane tension to gravity in an approximate cell model

**DOI:** 10.1186/s12976-019-0116-2

**Published:** 2019-12-05

**Authors:** Lili Wang, Weiyi Chen, Hongmei Guo, Airong Qian

**Affiliations:** 1 Shanxi Key Laboratory of Material Strength & Structural Impact, College of Biomedical Engineering, Taiyuan University of Technology, Taiyuan, 030024 China; 2 National Demonstration Center for Experimental Mechanics Education (Taiyuan university of Technology), Taiyuan, 030024 China; 3 Key Laboratory for Space Biosciences & Biotechnology, Faculty of Life Sciences, Northwestern Polytechnical University, Xi’an, 710072 China

**Keywords:** Gravity, Equilibrium differential equation, Membrane tension, Pseudo-ellipsoidal cap, Pseudo-spherical cap

## Abstract

**Background:**

Gravity, especially hypergravity, can affect the morphology of membranes, and further influence most biological processes. Since vesicle structures are relatively simple, the vesicle can be treated as a vital model to study the mechanical properties of membranes in most cases. Basic research on membrane tension has become a vital research topic in cellular biomechanics.

**Methods:**

In this study, a new vesicle model is proposed to quantitatively investigate the response of membrane tension to gravity. In the model, the aqueous lumen inside the vesicle is represented by water, and the vesicle membrane is simplified as a closed, thin, linear elastic shell. Then, the corresponding static equilibrium differential equations of membrane tension are established, and the analytical expression is obtained by the semi-inverse method. The model parameters of the equations are accurately obtained by fitting the reported data, and the values calculated by the model agree well with the reported results.

**Results:**

The results are as follows: First, both the pseudo-ellipsoidal cap and the pseudo-spherical cap can be used to describe the deformed vesicle model; however, the former can better represent the deformation of the vesicle model because the variance of the pseudo-ellipsoidal cap is smaller. Second, the value of membrane tension is no longer a constant for both models. Interestingly, it varies with the vesicle height under the action of gravity. The closer it is to the substrate, the greater the membrane tension. Finally, the inclination between the tangent and the radial lines at a certain point is nearly proportional to the radius of the cross section in both models.

**Conclusion:**

These findings may be helpful to study the vesicle model spreading more accurately by taking into account the influence of gravity because it could affect the distribution of membrane tension. Furthermore, it may also provide some guidance for cell spreading and may have some implications for membrane tension-related mechanobiology studies, especially in the hypergravity conditions.

## Background

Gravity is constantly exerted on organisms [1], and some studies have shown that gravity can affect numerous physical and biological processes: biological cells are no exception [2]. Biological systems interact with gravity on different levels of organization, from the whole organisms [3] to cells [4], to membranes [5] and even down to the function of single proteins [6], and many experiments have directly demonstrated that biological processes from single molecules to various levels of tissue are dependent on gravity. For example, Sieber et al. indicated that the viscosity, conductance and capacity of membranes are dependent on gravity [2, 7]. Häder D. et al. showed that single cells can sense gravity [8].

Cell mechanical stimulations include mechanical stretch, compression, hydrostatic pressure, microgravity and hypergravity. Microgravity is the absence of gravity, which usually exists in spaceflights to different planets or moons. Hypergravity, which may be experienced by living cells in certain planets and during human highly accelerated flights, refers to conditions that have greater gravitational force than the gravity of the earth [9]. For example, astronauts are transiently affected by hypergravity during the processes of launching and returning to Earth, and military pilots are subject to hypergravity when they are engaged in certain sports, such as motor racing, motorcycling, and bobsledding [1]. Moreover, some studies have shown that hypergravity can affect behaviors of cells, such as proliferation [10, 11], gene expression [12], differentiation [13], development and apoptosis [14], morphology and function [1, 15], and cytoskeletal reorganization, adhesion and movement [16].

The mechanical properties of membranes can affect most biological processes, and membrane tension is a basic physical parameter of membranes that is involved in various biological processes, such as membrane trafficking, cell shape, adhesion, growth, endocytosis and motility [17, 18]. Some studies have emphasized the importance and contributions of membrane tension in biological processes [19–23], and have shown that membrane tensions originate mainly from the hydrostatic pressure across the lipid bilayer and cytoskeleton (CSK)-membrane adhesion [22]. However, Reinhart-King et al. emphasized that cells can exert significant forces before complete actin polymerization or visible stress fibre formation, which means that vesicles can be used to model cell adhesion [24]. In addition, Liu et al. indicated that the complexity of cells could be avoided by using the vesicles as a biomimetic model of cells since there is no CSK or nucleus in the vesicles, and emphasized that a fully three-dimensional (3D) model of a vesicle could be used to simulate a real cell [25]. Lu et al. have also shown that vesicle models are a most useful tool to explore the relevant issues [26].

In summary, due to the high costs and limited number of experiments in real microgravity, and the fact that there is little research on the response of membrane tension to gravity, this paper applies a theoretical modelling method to study the response of membrane tension under the action of gravity. In the model, the vesicles are simplified as water sacs, where the membrane of the vesicle is assumed to be a thin closed shell and the vesicle cavity is represented by water. The equilibrium differential equations of the deformed vesicle model are constructed and solved by using the analysis method of the elasticity mechanics and semi-inverse method.

## Methods

In this paper, a vesicle model is used to quantitatively investigate the response of membrane tension to gravity. In the vesicle model, the water represents aqueous lumen inside the vesicle and a elastic shell represents the membrane of vesicle. The corresponding static equilibrium differential equations of membrane tension are established by theoretical method. The model parameters of the equations are accurately obtained by fitting the reported data, and the analytical expression is obtained by the semi-inverse method.

### Hypotheses

Since vesicles are spherical structures consisting of a single bilayer (i.e., membrane) surrounding an aqueous lumen [26] and lipid bilayers are natural mimics of cellular membranes, vesicles are commonly used as stable model systems for studying numerous biological processes, such as, the adhesion of vesicles [27], membrane protein behaviour [28], the mechanism of cell endocytosis [29], membrane vesicle budding [30], and changes in membrane morphology [31]. In this study, considering the complexity of the cell structures, such as eukaryotic cell, the vesicle structures are relatively simple and often used to simulate cells, and a 3D axisymmetric model of vesicles is established to study the response of membrane tension to gravity. For simplicity, the following assumptions are made:

- The vesicles are simplified into watery sacs according to the structure of vesicles [26].
- In the model, the water representing the cavity of the vesicle is wrapped with a membrane, and its volume remains constant [25].
- The details of the molecular structure within the vesicle membrane are ignored [27], and the membrane is assumed to be isotropic, linearly elastic, of fixed thickness, thin and a closed shell [25].
- The un-deformed vesicle model is used to represent the suspended cell described by the sphere [32]. Since the cells gradually switch from a spherical to a flattened shape in vitro [33], the spread cell is described by the deformed vesicle model under the action of gravity.
- The deformation of the vesicle model is assumed to be generated under the conditions of axial symmetry, and the contact area is always circular [34].

### Equilibrium equations

**Fig. 1:**
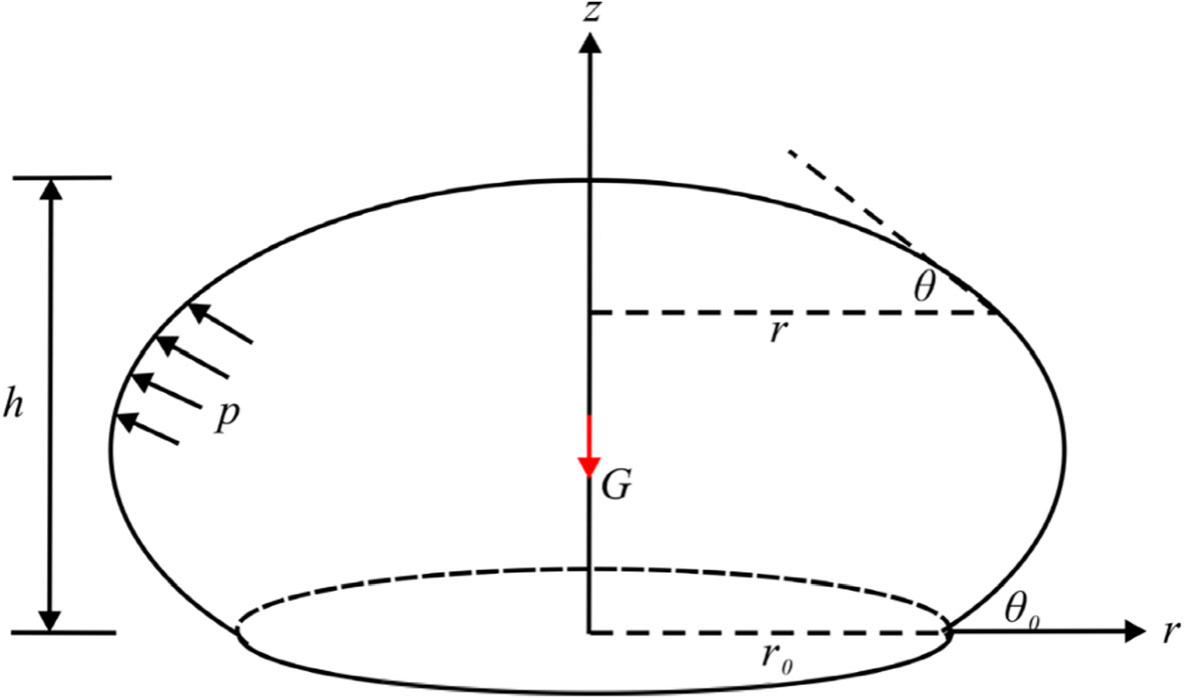
Schematic diagram of the deformed vesicle model**:** *G* is gravity applied to the centroid and an internal pressure *p* acts on the membrane wall. *r* is the radial distance of any point on the free part of the vesicle model and *θ* is the inclination at the same point. The contact area is circular, and *r*_0_ is the contact radius. *θ*_0_ denotes the contact angle, and *h* is the height of the vesicle model

In the model, the cylindrical coordinate (*r,* *z*) is used in which the *z* axis is directed against the gravity force and *r* is an independent variable. The local slope of the membrane at the same point is defined by the inclination *θ* between the tangent line and the radial line as illustrated in Fig. 1.

The two variables *z*(*r*) and *θ*(*r*) are related through [35]:

2.1 $$ \frac{dz}{dr}={z}^{\prime }(r)=-\tan \theta $$

**Fig. 2:**
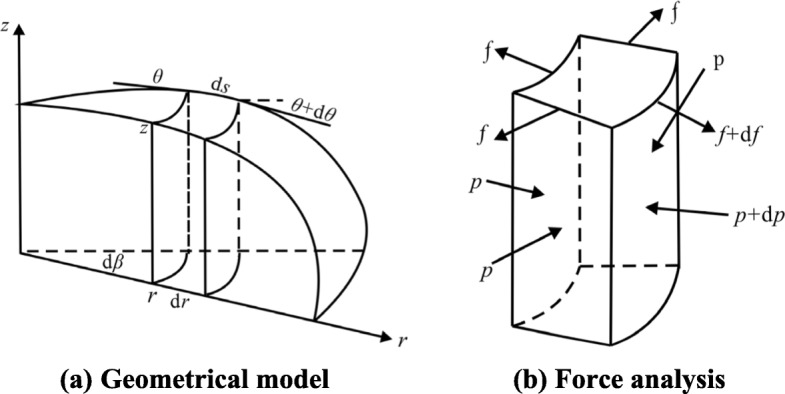
Schematic diagram of the geometrical model of the deformed vesicle and its force balance in the *r*-direction: **a** A geometrical model of the deformed watery sac: a micro-block is taken from the deformed vesicle model. The radius of the micro-block position is *r*, the height is *z*, the thickness is d*r*, the angle of rotation is d*β*, and the arc length of the micro-block is d*s*. **b** A force diagram of the micro-block, where *f* is membrane tension

Consider the equilibrium of the deformed vesicle model in the *r*-direction for a micro-block between *r* and *r* + d*r*; the force analysis diagram is shown in Fig. 2.

From the equilibrium condition in the *r*-direction, the formulation is established as follows:

2.2a $$ {\displaystyle \begin{array}{l}- frd\beta \cos \theta +\left(f+ d f\right)\left(r+ d r\right) d\beta \cos \left(\theta + d\theta \right)-2 fds\sin \frac{d\beta}{2}\\ {}+ przd\beta -\left(p+ d p\right)\left(r+ d r\right)\left(z+ d z\right) d\beta +2 pzdr\sin \frac{d\beta}{2}=0\end{array}} $$

Because d*θ* and d*α* are small quantities, the formulation is simplified:

2.2b $$ \sin \frac{d\theta}{2}\approx \frac{d\theta}{2},\cos \frac{d\theta}{2}\approx 1 $$

By simplifying and neglecting the small quantities of higher order, the equilibrium differential equation is obtained in the *r*-direction:

2.2c $$ \frac{f}{r}-f\tan \theta {\theta}^{\prime }-\frac{f{s}^{\prime }}{r\cos \theta }+{f}^{\prime }=\frac{1}{\cos \theta}\left(p{z}^{\prime }-{p}^{\prime }z\right) $$

**Fig. 3:**
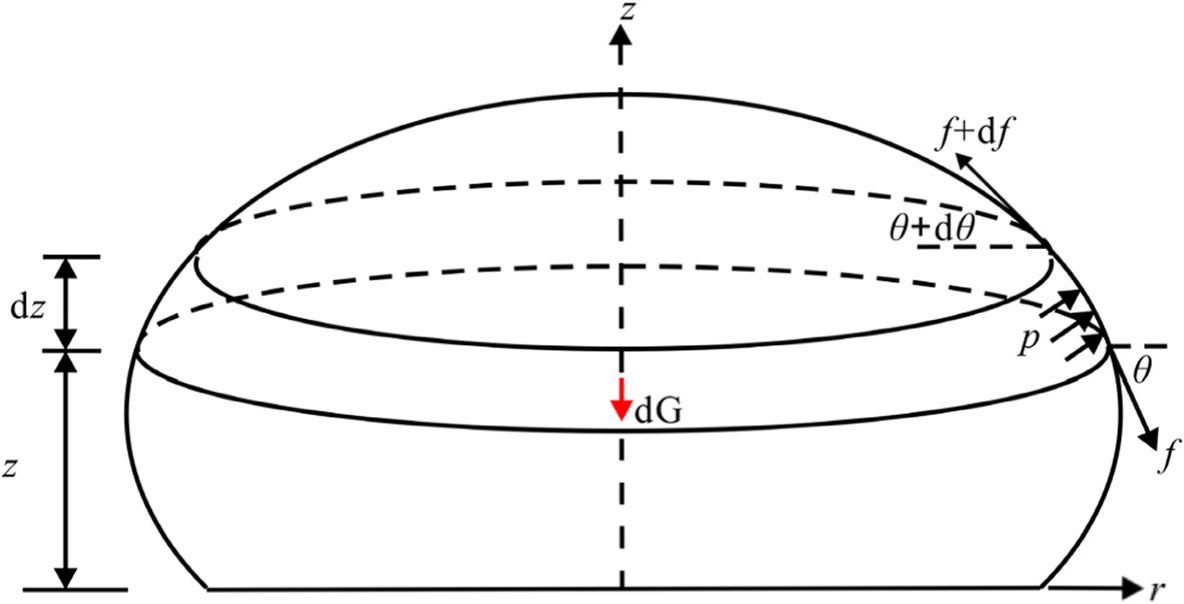
Schematic diagram of force balance in the *z*-direction for the deformed vesicle model

In a similar manner, the equilibrium differential equation in the *z*-direction for a slice between *z* and *z* + d*z* can be obtained as follows. The force analysis diagram is shown in Fig. 3.

From the equilibrium condition in the *z*-direction:

2.3a $$ {\displaystyle \begin{array}{l}- dG+p\cdot \pi {r}^2-\left(p+ d p\right)\cdot \pi {\left(r+ d r\right)}^2-f\cdot 2\pi r\cdot \sin \theta \\ {}+\left(f+ d f\right)\cdot 2\pi \left(r+ d r\right)\cdot \sin \left(\theta + d\theta \right)=0\end{array}} $$

and simply:

2.3b $$ f\cdot {\theta}^{\prime }+\frac{f}{r}\tan \theta +{f}^{\prime}\cdot \tan \theta =\frac{p}{\cos \theta } $$

By solving the system of eqs. (2.1~2.3), the analytic expression of membrane tension is obtained:

2.4 $$ f=\frac{\frac{p}{\tan \theta }-p{z}^{\prime }+{p}^{\prime }z}{\frac{\theta^{\prime }}{\sin \theta }{\cos}^2\theta +\sin \theta {\theta}^{\prime }+\frac{s^{\prime }}{r}} $$

In the above equations, the internal pressure *p* meets the following relationship:

2.5 $$ p=p(z)={p}_0-\rho gz $$

where *p*_0_ is the bottom pressure.

### Boundary conditions

It has been shown that the solution may be reduced to solve the differential equations of the equilibrium together with the boundary conditions. To solve the above equations, the global equilibrium condition of the membrane is required:

2.6 $$ 2\pi {r}_0{F}_0\sin {\theta}_0=\pi {r_0}^2{p}_0-G $$

where *F*_0_ is the bottom membrane tension determined by using the classic wetting formula of Young’s Eq. (2.7) [34], in which, *θ*_0_ is the contact angle.

2.7 $$ \varGamma ={F}_0\left(1-\cos {\theta}_0\right) $$

## Equation solving

In this study, the semi-inverse method is used. In the semi-inverse method, one guesses parts of the solution and then tries to determine the rest rationally so that all of the differential equations and boundary conditions are satisfied. As we know, the guessed solution is an exact solution of this problem. In view of the abovementioned facts, we assume the shapes of the deformed vesicles model, then obtain the solutions of membrane tension.

### Pseudo-ellipsoidal cap

**Fig. 4:**
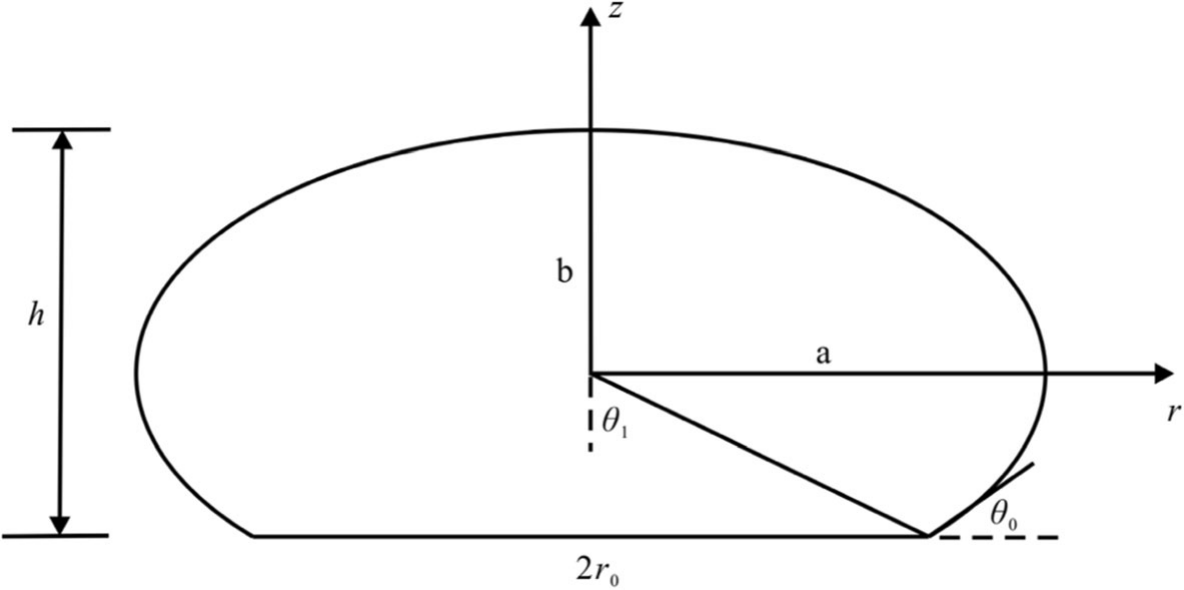
Schematic diagram of an ellipsoidal cap representing the deformed vesicle model on a supporting solid surface

In the model, the un-deformed vesicle is denoted by the sphere, and the deformed vesicle is represented by the ellipsoidal cap obtained by rotating the ellipse about the *z* axis, as shown in Fig. 4.

The ellipsoidal cap geometry is a three-parameter model defined in terms of contact radius *r*_0_, height *h* and contact angle *θ*_0_. All three measured quantities are required to evaluate the volume of the ellipsoidal cap. In the *r-z* plane, the geometry profile of the vesicle model is an ellipse described by the equation

3.1 $$ \frac{r^2}{a^2}+\frac{z^2}{b^2}=1 $$

where, *a* and *b* are the semi-axis lengths in the *r* and *z* directions, respectively. The values of *a* = 16.67 μm and *b* = 15.90 *μm* are obtained by fitting the experimental data in Table 1. The fitting method used in this paper is the polynomial fitting in Origin 8.5. In the fitting process, the contact radius *r*_0_ is obtained according to the bottom area in Table 1, and then the values of the semi-axis lengths *a* and *b* is obtained by fitting the contact radius *r*_0_ and height *h* using Eq. (3.1).

**Table 1:** Dimensions of different spreading states [36]

Different spreading states	Bottom area (*μm*^2^)	Height (*μm*)	Volume(*μm*^3^)
a	125.09	15.45	2988.40
b	243.84	12.75	2998.36
c	421.28	10.96	3000.60
d	549.88	9.41	3000.49
e	696.53	8.03	3010.90

In the ellipse, the relationship between angles *θ*_1_ and *θ*_0_ satisfies the eq. (3.2),and *ε*_*r*_ is the ratio of the axes, *ε*_*r*_ = *b*/*a* [37].

3.2 $$ \tan {\theta}_0={\varepsilon}_r^2\tan {\theta}_1={\varepsilon}_r^2\frac{r_0}{b-h}=\frac{2h{r}_0}{{r_0}^2-{\left(\frac{h}{\varepsilon_r}\right)}^2} $$

The volume of the ellipsoidal cap can be obtained [38]

3.3 $$ V=\frac{\pi {r}_0h}{3}\frac{\left(2{r}_0\tan {\theta}_0-h\right)}{\tan {\theta}_0} $$

The differential surface area of the ellipsoidal cap can be obtained

3.4 $$ dA=\left(\raisebox{1ex}{$2\pi a$}\!\left/ \!\raisebox{-1ex}{${b}^2$}\right.\right)\sqrt{b^4+\left({a}^2-{b}^2\right){z}^2} dz $$

The heights determined by the geometric equation Eq. (3.1) cannot rigorously satisfy the volume formula Eq. (3.3). To overcome this deficiency, a geometry of the pseudo-ellipsoidal cap is offered using the correction parameter *m,* as shown below:

3.5 $$ \tan {\theta}_0={\varepsilon}_r^2\frac{r_0}{b- mh}=\frac{2 mh{r}_0}{r_0^2-{\left(\frac{mh}{\varepsilon_r}\right)}^2} $$

By combining Eq. (3.3) and Eq. (3.5), the volume of the pseudo-ellipsoidal cap is obtained

3.6 $$ V=\frac{\pi mh}{6{\varepsilon}_r^2}\left[3{r}_0^2{\varepsilon}_r^2+{m}^2{h}^2\right] $$

Then, the analytic expression of membrane tension is formulated:

3.7 $$ f=\frac{\sqrt{a^4{m}^2-{a}^2{m}^2{r}^2+{b}^2{r}^2}\left[{p}_0\left({a}^4{m}^2-{a}^2{m}^2{r}^2+{b}^2{r}^2\right)-\rho gabm{\left({a}^2-{r}^2\right)}^{3/2}\right]}{b\left(2{a}^4{m}^2-{a}^2{m}^2{r}^2+{b}^2{r}^2\right)} $$

### Pseudo-spherical cap

**Fig. 5:**
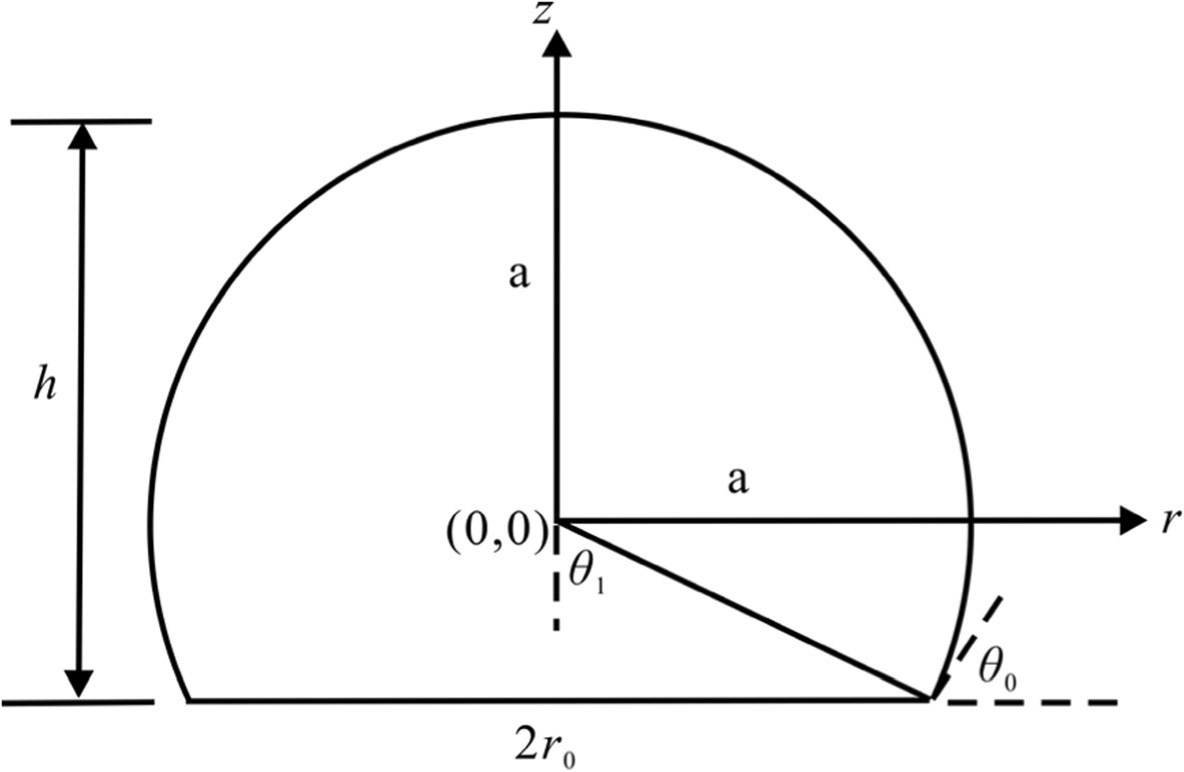
Schematic diagram of the spherical cap

Since the deformed cells are represented by the spherical cap [32], a pseudo-spherical cap is selected to simulate the deformed vesicle model more accurately. In the pseudo-ellipsoidal cap model, when the ratio of the axes *ε*_r_ is equal to 1, a pseudo-spherical cap is formed; when the correction parameter *m* is 1, the spherical cap is generated, as shown in Fig. 5.

The radius of the spherical cap *R* = *a* = *b* = 16.26 μm is obtained by fitting the experimental data in Table 1. Similarly, the heights determined by the geometric equation cannot completely meet the volume formula, and the correction parameter *m* is needed. In the pseudo-spherical cap, the relationship between the height *h* and contact radius *r*_0_ satisfies [39]:

3.8 $$ mh={r}_0\tan \frac{\theta_0}{2} $$

It yields the following relationship between the height *h* and the contact radius *r*_0_ under the condition without the volume dilatation:

3.9 $$ V=\frac{\pi mh}{6}\left[3{r_0}^2+{m}^2{h}^2\right] $$

The surface area of the pseudo-spherical cap can be obtained:

3.10 $$ A=2\pi Rmh $$

In the pseudo-spherical model, the analytic expression of membrane tension is expressed as:

3.11 $$ f=\frac{\sqrt{a^2{m}^2-{m}^2{r}^2+{r}^2}\left[{p}_0\left({a}^4{m}^2-{a}^2{m}^2{r}^2+{a}^2{r}^2\right)-\rho g{a}^2m{\left({a}^2-{r}^2\right)}^{3/2}\right]}{\left(2{a}^4{m}^2-{a}^2{m}^2{r}^2+{a}^2{r}^2\right)} $$

### Analytical approximation

The total energy *U*_T_ of this system is made up of three terms: the elastic energy of the membrane *U*_E_, the mechanical energy of gravity *U*_G_ and the surface energy *U*_S_ [40].

3.12 $$ {U}_T={U}_E+{U}_G+{U}_S $$

In this study, the single variable method is adopted, so the fluidity and viscosity of the liquid are ignored. The deformation of the vesicle model is primarily due to gravity. A spherical cap is selected to describe the geometry of the deformed vesicle model and the initial bending energy of the un-deformed vesicle model is set to 0. Then, the following equation can be obtained:

3.13 $$ \frac{U_S}{U_E+{U}_G}=\frac{-\pi {r_0}^2\varGamma }{G\left[{R}_0+R- mh\right]+\frac{\pi \kappa mh}{R}+ f\varDelta A} $$

For representative parameter values *κ* = 20*k*_*B*_*T* ≈ 10^− 19^ *J* [34], *mh/R* = 1.8, *R*_0_ = 8.9 μm volume of approximately 3000 *μm*^3^ [41], *f* ≈ 4 × 10^− 4^ *N/m* and *G* = *ρgV* = 29.4 × 10^− 12^ *N*, the value of this ratio is approximately 10^8^. For this reason, the elastic energy of the membrane and the work performed by gravity are ignored throughout the analysis, and the contact radius is determined by the following equation first solved by Johnson, Kendall, and Roberts (JKR theory) [39, 42].

3.14 $$ {r}^3=\frac{9\pi \left(1-{\nu}^2\right)}{2E}{R}_0^2\varGamma $$

In this article, the Young’s modulus *E* and the Poisson ratio *ν* of the membrane are 1000 *Pa* and 0.3, respectively [36]. The membrane thickness *t* is 0.1 *μm* [43]. When the adhesion energy per unit area *Г* is chosen to be 6 × 10^− 4^ *J/m*^*2*^ [33], *r*_0_ is 8.49 *μm*.

In the pseudo-ellipsoidal cap model, the height *h* is calculated to be 13.29 *μm,* and the correction parameter *m* is equal to 1.027, according to Eq. (3.1) and Eq. (3.6). The contact angle *θ*_0_ is 73.75° by application of Eq. (3.5). The tension *F*_0_ is 0.83 *mN/m* by using Eq. (2.7), and *p*_0_ is equal to 188.52 *Pa* by application of Eq. (2.6).

Furthermore, in the pseudo-spherical situation, the height *h* is 13.02 *μm* and the correction parameter *m* is equal to 1.071 using Eq. (3.9). The contact angle *θ*_0_ is 62.54° by using Eq. (3.8). The tension *F*_0_ is 0.41 *mN/m,* and *p*_0_ is 86.14 *Pa*.

## Results and discussion

**Fig. 6:**
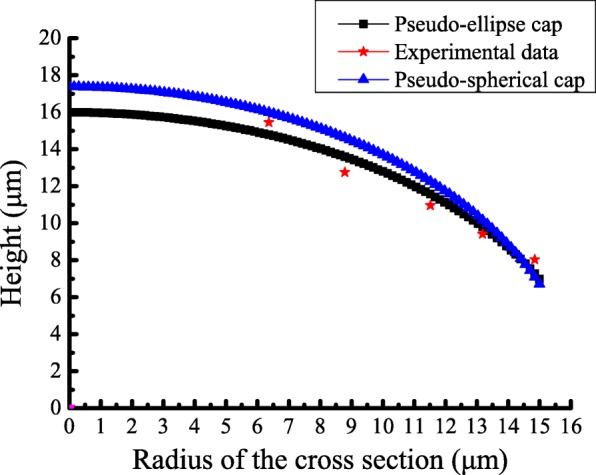
Curve of the height *h* against the radius of the cross section *r*

Vesicle model deformation from the spherical state to the pseudo-ellipsoidal cap state under the action of gravity is a quasi-static process. The curve of the height *h* against the radius of the cross section *r* is shown in Fig. 6. The height *h* decreases when the radius of the cross section *r* increases, which agrees well with the experimental data. The results indicate that both the pseudo-ellipsoidal cap and the pseudo-spherical cap can describe the deformed vesicle model by gravity. To evaluate which can better represent the geometry of the deformed vesicle model, the variance formula is used to estimate the errors. The variance of the pseudo-ellipsoidal cap is equal to 0.83, and that of the pseudo-spherical cap is 1.23, indicating that the pseudo-ellipsoidal cap may be a better representation of the deformed vesicle model.

**Fig. 7:**
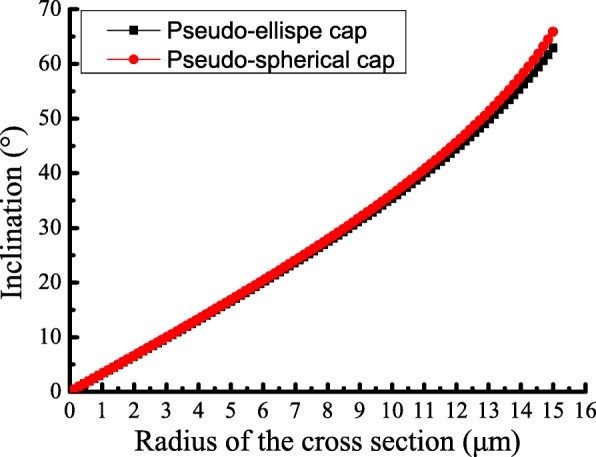
Curve of the inclination *θ* against the radius of the cross section *r*

The relationship between the inclination *θ* (*θ* < 90°) and the radius of the cross section *r* is obtained as shown in Fig. 7. The result shows that the inclination *θ* is positively correlated with the radius of the cross section *r*. The angle gradually increases as the radius of the cross section *r* increases. The values of the contact angle *θ*_0_ are 62.43° and 65.86°, respectively. This means that the different models are chosen to describe the vesicle model deformations; however, the results of the relationship between the inclination *θ* and the radius of the cross section *r* are the same results as shown in Fig. 7.

**Fig. 8:**
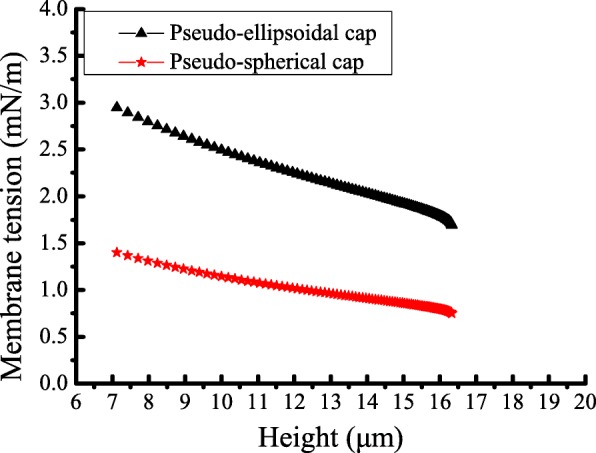
Curves of the membrane tension against the height

Moreover, the variation of membrane tension *f* with height *h* is also analysed as shown in Fig. 8. The results show that membrane tension increases with decreasing height *h*; however, the value of the pseudo-ellipsoidal cap is slightly larger than that of the pseudo-spherical cap. In the pseudo-ellipsoidal cap, the minimum and maximum values of membrane tension are 1.69 *mN/m* and 2.95 *mN/m*, respectively, while the values in the pseudo- spherical cap are 0.75 *mN/m* and 1.40 *mN/m*, respectively. This suggests that the membrane tension of the former is approximately twice that of the latter, which may be caused by the bottom pressure. Nevertheless, in both models, membrane tensions are slightly larger than the reported values, which are approximately 0.2~0.4 *mN/m*. This means that gravity may have a slight influence on membrane tension when using the single variable method, so considering gravity may contribute to more accurate study of the spreading of vesicles model.

From the above, a macro approximation is used to describe the deformation of the vesicle model under the action of gravity in the present study. It can be used to quantitatively describe the response of membrane tension to gravity. Furthermore, the rationality of using the pseudo-ellipsoidal cap and pseudo-spherical cap to represent the deformed vesicle model is explained from a mathematical point of view.

However, there are some obvious deficiencies in this study. Firstly, due to the very complex structure of eukaryotic cells, the proposed vesicle model may not be suitable for studying the eukaryotic cells. Since some studies have shown that the behavior of bacterial, the ability of bactetia to sense the surrounding environment can change under microgravity, and intestinal microbes can be dysregulated in microgravity. While bacterial behavior can affect manned spaceflight, and the intestinal microbial disorders can lead to a series of diseases [44]. Given the wide variety of cell types, the relatively simple structure of vesicles and the ability of vesicles to mimic cells, this model can be used to study the changes of prokaryotic cells without CSK under the action of microgravity, such as bacteria. In addition, the model can be also used to study the changes of membrane tension in simple cells, such as membrane tension variation of red blood cells, which are subjected to fluid shear stress in blood vessels [45]. Secondly, when studying the response of membrane tension to gravity, the single variable method is used, and the fluidity and viscosity of the liquid are ignored, which seems to be slightly different from the actual situation. In the future, we will comprehensively study the variations of membrane tension with height under the action of gravity, liquid fluidity and viscosity, and study whether gravity has a significant influence on the magnitude and distribution of membrane tension compared with other factors.

## Conclusions

To summarize, a theoretical model of the deformation of the vesicle under the action of gravity is developed to study the response of membrane tension to gravity. The equilibrium differential equations, mainly consisting of gravity, internal pressure and membrane tension, are established. The analytic expression of membrane tension is obtained. Our findings can be succinctly summarized as follows:

- The deformed geometry of the vesicle model can be represented by both the pseudo-ellipsoidal cap and the pseudo-spherical cap under the action of gravity, and the pseudo-ellipsoidal cap is better from a mathematical point of view.
- The membrane tension varies with the height: the closer it is to the basement, the greater the membrane tension.
- The inclination *θ* between the tangent line and radial line is nearly proportional to the radius of the cross section *r* in both models.
- Considering gravity may be useful to more accurately study the spreading of the vesicle model since gravity can influence the distribution of membrane tension.

The focus of the present work is to quantitatively analyse the response of membrane tension to gravity. These findings may provide certain guidance for cell model spreading and may have some implications for membrane tension-related biological processes, especially under the hypergravity conditions.

## Data Availability

All data used and analyzed during the current study available from the corresponding author on reasonable request.

## Abbreviations

3D: three dimensions
*a*: major semi axes of ellipse cap (μm)
*b*: minor semi axes of ellipse cap (*μm*)
CSK: cytoskeleton
*E*: elasticity modulus of membrane (*Pa*)
*f*: membrane tension (*N/m*)
*F*_0_: bottom membrane tension *(N/m*)
*f*_0_: initial membrane tension (*N/m*)
*G*: gravity (*N*)
*h*: height of deformed vesicle (μm)
*k*_*B*_: boltzmann constant
*m*: correction parameter
*p*_0_: bottom pressure (*Pa*)
*r*: radial distance of any point (μm)
*R*: radius of spherical cap (*μm*)
*r*_0_: contact radius (*μm*)
*R*_0_: radius of spherical vesicle (*μm*)
*T*: absolute temperature
*t*: thickness of membrane (*μm*)
*U*_*E*_: elastic energy of membrane (*J*)
*U*_*G*_: mechanical energy done by gravity (*J*)
*U*_*S*_: surface energy (*J*)
*U*_*T*_: total energy of vesicle (*J*)
*V*: volume of vesicle (*μm*^3^)
*ε*_*r*_: ratio of minor semi axes *b* and major semi axes *a* of ellipse cap
*θ*: angle of any point between the tangent line and the radial line (°)
*θ*_0_: contact angle (°)
*κ*: bending rigidity of membrane (*J*)
*ν*: Poisson ratio of membrane
*ρ*: density of water (*kg/m*^3^)
*Г*: adhesion energy per unit area (*J/m*^2^)
